# Sex Differences on Clinical Characteristics, Severity, and Mortality in Adult Patients With COVID-19: A Multicentre Retrospective Study

**DOI:** 10.3389/fmed.2021.607059

**Published:** 2021-02-12

**Authors:** Jing Sha, Guoqiang Qie, Qingchun Yao, Wenqing Sun, Cuiyan Wang, Zhongfa Zhang, Xingguang Wang, Peng Wang, Jinjiao Jiang, Xue Bai, Yufeng Chu, Mei Meng

**Affiliations:** ^1^Department of Critical Care Medicine, Shandong Provincial Hospital Affiliated to Shandong First Medical University, Shandong Provincial Hospital Affiliated to Shandong University, Shandong University, Jinan, China; ^2^Department of Intensive Care Unit, Shandong Provincial Chest Hospital, Jinan, China; ^3^Shandong Medical Imaging Research Institute Affiliated to Shandong University, Jinan, China; ^4^Jinan Infectious Diseases Hospital, Shandong University, Jinan, China; ^5^Department of Pulmonary and Critical Care Medicine, Shandong Provincial Hospital Affiliated to Shandong First Medical University, Jinan, China; ^6^Department of Critical Care Medicine, Ruijin Hospital, Ruijin Hospital North, Shanghai Jiao Tong University School of Medicine, Shanghai, China

**Keywords:** COVID-19, sex, estrogen, menopause, mortality, China

## Abstract

**Background:** Coronavirus disease-2019 (COVID-19) epidemic is spreading globally. Sex differences in the severity and mortality of COVID-19 emerged. This study aims to describe the impact of sex on outcomes in COVOD-19 with a special focus on the effect of estrogen.

**Methods:** We performed a retrospective cohort study which included 413 patients (230 males and 183 females) with COVID-19 from three designated hospitals in China with a follow up time from January 31, 2020, to April 17, 2020. Women over 55 were considered as postmenopausal patients according to the previous epidemiological data from China. The interaction between age and sex on in-hospital mortality was determined through Cox regression analysis. In addition, multivariate Cox regression models were performed to explore risk factors associated with in-hospital mortality of COVID-19.

**Results:** Age and sex had significant interaction for the in-hospital mortality (*P* < 0.001). Multivariate Cox regression showed that age (HR 1.041, 95% CI 1.009–1.073, *P* = 0.012), male sex (HR 2.033, 95% CI 1.007–2.098, *P* = 0.010), the interaction between age and sex (HR 1.118, 95% CI 1.003–1.232, *P* = 0.018), and comorbidities (HR 9.845, 95% CI 2.280–42.520, *P* = 0.002) were independently associated with in-hospital mortality of COVID-19 patients. In this multicentre study, female experienced a lower fatality for COVID-19 than male (4.4 vs. 10.0%, *P* = 0.031). Interestingly, stratification by age group revealed no difference in-hospital mortality was noted in women under 55 compared with women over 55 (3.8 vs. 5.2%, *P* = 0.144), as well as in women under 55 compared with the same age men (3.8 vs. 4.0%, *P* = 0.918). However, there was significantly difference in women over 55 with men of the same age group (5.2 vs. 21.0%, *P* = 0.007). Compared with male patients, female patients had higher lymphocyte (*P* < 0.001) and high-density lipoprotein (*P* < 0.001), lower high sensitive c reaction protein level (*P* < 0.001), and lower incidence rate of acute cardiac injury (6.6 vs. 13.5%, *P* = 0.022).

**Conclusion:** Male sex is an independent risk factor for COVID-19 in-hospital mortality. Although female mortality in COVID-19 is lower than male, it might not be directly related to the effect of estrogen. Further study is warranted to identify the sex difference in COVID-19 and mechanisms involved.

## Introduction

The whole world is currently under the effect of the ongoing epidemic of coronavirus disease-2019 (COVID-19), caused by a novel coronavirus termed severe acute respiratory syndrome coronavirus (SARS-CoV-2). As of 17 June 2020, the World Health Organization (WHO) has reported 8,061,520 confirmed cases and 440,290 deaths in 216 countries and regions, and COVID-19 has become a public health emergency of international concern (PHEIC) (1). Although most of the COVID-19 patients are non-severe and self-limited, there are still 16% severe cases and 3.1% died in China (2–5).

The current studies showed that advanced age and comorbidities were closely related to worse prognosis (6–9). Recently a retrospective multicentre cohort study demonstrated older COVID-19 patients tended to have relatively more severe clinical infections and poorer clinical outcomes associated with COVID-19 compared with younger patients in Jiangsu of China (10). In addition, in a multicentre Italian CORIST study including 3,894 patients with SARS-CoV-2 infection found advanced age at hospital admission was one of powerful predictors of higher in-hospital death (11). Meanwhile, evidence of sex differences in COVID-19 severity emerged, where the morbidity and mortality were all higher among males than females (12–14). A male bias in COVID-19 mortality was reported in 37 of the 38 countries that have provided sex-disaggregated data. Scully et al. showed that the average male case fatality rate (CFR) across 38 countries was 1.7 times higher than the average female CFR (15). Previous epidemiological studies showed the proportion of individuals infected and CFRs in severe acute respiratory syndrome (SARS)-CoV and Middle East respiratory syndrome (MERS)-CoV were higher in males than that of females (16, 17). The causes of Sex differences following virus infections are multifactorial, including differences in steroid hormones, immune response X-linked genes, disease-susceptibility genes in sexes, and gender-related social factors (18–20).

Studies have linked increased susceptibility to infection with circulating steroid hormone concentrations (18, 19, 21). Traditionally, sex steroid hormones, especially the estrogen in females, have been considered for their immunomodulatory properties. Animal study indicated that ovariectomy or treating female mice with estrogen receptor antagonist increased mortality, indicating a protective effect for estrogen receptor signaling in mice infected with SARS-CoV (22). Thus, the lower incidence of severe COVID-19 in female patients might be related to the protective effect of estrogen. However, as yet, few studies have focused on sex differences in clinical characteristics and laboratory tests of COVID-19, especially whether estrogen affects the occurrence and development of COVID-19. Therefore, this study aims to analysis the sex differences on clinical characteristics, severity and mortality in adult patients with COVID-19, and explore possible mechanisms, with a special focus on premenopausal and postmenopausal women.

## Materials and Methods

### Study Design and Participants

The multicentre retrospective cohort study was conducted at three hospitals designated for the treatment of COVID-19, including Jinan Infectious diseases Hospital in Shandong, Shandong Provincial Chest Hospital in Shandong, and Huanggang Central Hospital in Hubei. The recruitment period was from January 31, 2020, to April 17, 2020. The diagnosis of COVID-19 was made based on the National Health Commission of China guidance (23). The presence of SARS-COV-2 in respiratory specimens was confirmed using real-time reverse-transcriptase polymerase chain reaction (RT-PCR) assay were performed in accordance with the protocol described previously (2). The patients that are pregnant or <18 years old were excluded. As of April 17, 2020, all included patients were discharged or died. In addition, the vast majority of city women were postmenopausal by age 55 (24, 25), according to the previous epidemiological data from China, which was consistent with our study. Thus, patients were divided into two groups according to the age of 55 to explore the role of estrogen in the progression of COVID-19. The study was approved by the institutional review board of Jinan Infectious diseases Hospital, Shandong Provincial Chest Hospital, and Huanggang Central Hospital.

### Data Collection

Two physicians reviewed clinical electronic medical records and laboratory findings for all patients with SARS-CoV-2 infection, and then a third researcher determined any differences between interpretations of the two primary reviewers. The demographic data, menstrual history of women, clinical characteristics and laboratory results were collected at admission. We also evaluated and gathered complications, treatment and clinical outcomes (discharged alive or dead) at the end of study, by using a standardized case-report form. For patients with a readmission during the study period, data from the first admission were presented. Sequential Organ Failure Assessment (SOFA) scores were calculated using the worst value of physiological variables within 24 h of presentation.

### Diagnostic and Grading Criteria for COVID-19

The disease severity of COVID-19 patients was divided into severe and non-severe conditions, defined according to the American Thoracic Society guidelines for community-acquired pneumonia (26). Severe COVID-19 should reach the following either one major criterion or three or more minor criteria. In detail, Minor criteria included respiratory rate more than 30 breaths per minute, PaO2/FIO2 ratio lower than 250, multilobar infiltrates confusion or disorientation, blood urea nitrogen level more than 7.1 mmol/L, white blood cell count <4.0 × 10^9^ per L, platelet count <100 × 10^12^ per L, core temperature lower than 36°C, and hypotension requiring aggressive fluid resuscitation. Major criteria included septic shock with need for vasopressors, or mechanical ventilation. Fever was defined as axillary temperature of at least 37.3°C. Septic shock was defined according to the 2016 Third International Consensus Definition for Sepsis and Septic Shock (27). Acute kidney injury was diagnosed according to the KDIGO clinical practice guidelines (28) and acute respiratory distress syndrome (ARDS) was diagnosed according to the Berlin Definition (29). Acute cardiac injury was diagnosed if serum levels of cardiac biomarkers (e.g., high-sensitive cardiac troponin I) were above the 99th percentile upper reference limit, or if new abnormalities were shown in electrocardiography and echocardiography (2).

#### Outcomes

The primary outcome was in-hospital mortality. The secondary outcomes were including disease severity, development of acute respiratory distress (ARDS), acute cardiac injury, acute liver injury, sepsis shock and acute kidney injury (AKI).

### Statistical Analysis

Patients were divided into two groups according to sex, and the subgroup analysis was performed at the cut-off point of 55 years old according to the age of menopause in women. Female patients and male patients were grouped by age into the younger group(less than or 55 years old) and the older group (above 55 years old) for comparison. Continuous variables were expressed as mean ± standard deviation (SD) or medians (interquartile range, IQR) values. Categorical data were summarized as frequency rates and percentages. The comparison between the two groups was conducted using *t*-tests or Mann-Whitney U tests for continuous variables, and chi-squared tests or Fisher's exact tests for categorical variables. The interaction between age and sex on in-hospital mortality was determined through Cox regression analysis. In addition, multivariate Cox regression models were performed to explore risk factors associated with in-hospital mortality of COVID-19. Considering the total number of death cases (*n* = 31) in this study and to avoid overfitting in the model, four factors with significant association with mortality in univariate regression analyses (sex, age, the interaction between age and sex, and comorbidities) were chosen for multivariate analysis on the basis of previous findings and clinical constraints (8, 10, 12, 13, 30). Hazard ratios (HRs) with 95% confidence intervals (CIs) and the corresponding *P* values were calculated for each risk factor. Kaplan-Meier estimator was generated to estimate the survival curves and log-rank test was used to compare the survival probability between male and female groups. *P* < 0.05 was considered statistically significant. The variables that had >5% of values missing were excluded. Simple data imputation was done for missing data <5%, using the median for skewed distribution data, or the mode for dichotomous data. All analyses were conducted with SPSS software, version 22.0 (SPSS Inc. Chicago, Illinois, United States).

## Results

### Basic Characteristics

A total of 441 COVID-19 patients (range, 2–89 years) were hospitalized in the three designated hospital from Jan 31, 2020 to Apr 17, 2020. After excluding one pregnant patient, 11 patients <18 years old, and 16 patients without available key information in their medical records, we included 413 patients in the final analysis, among whom 230 were males and 183 were females. Table 1 presented the sex-specific demographic and clinical characteristics of the all patients with COVID-19. The median age of all cases was 58 years old (IQR 47–67 years), and it had not difference between males and females. Comorbidities were common for both sexes, but no significant difference. Regarding the symptoms, fever, cough and chest distress were the most common on admission among both men and women. However, a higher percentage of men had fever (90.4% vs. 82.0%, *P* = 0.012). Additionally, SOFA score differed significantly between males and females.

**Table 1:** Sex-specific demographic and clinical characteristics of COVID-19 patients.

**Variables**	**Male (*n* = 230)**	**Female (*n* = 183)**	***P*-value**
Age, Median (IQR),y	56 (46, 67)	59 (49, 67)	0.094
current or ever smoking	18 (7.8)	0 (0)	**<0.001**
Drinking	17 (7.4)	0 (0)	**<0.001**
**Comorbidities**			
COPD	6 (2.6)	1 (0.5)	0.139
DM	30 (13.0)	24 (13.1)	0.983
Hypertension	70 (30.4)	47 (25.7)	0.287
Heart disease	15 (6.5)	11 (6.0)	0.832
Kidney disease	6 (2.6)	4 (2.2)	0.781
Liver disease	7 (3.0)	5 (2.7)	0.851
Shock	8 (3.5)	6 (3.3)	0.911
Tumor	6 (2.6)	7 (3.8)	0.482
Immune disease	4 (1.7)	4 (2.2)	0.744
**Symptoms**			
Fever	208 (90.4)	150 (82.0)	**0.012**
Cough	178 (77.4)	142 (76.1)	0.961
Expectoration	81 (35.2)	57 (31.1)	0.384
Chest distress	110 (47.8)	92 (50.2)	0.621
Chest pain	5 (2.2)	8 (4.4)	0.204
Hemoptysis	4 (1.7)	2 (1.1)	0.697
Headache	12 (5.2)	11 (6.0)	0.727
Myalgia	24 (10.4)	24 (13.1)	0.399
Fatigue	83 (36.1)	71 (38.8)	0.571
Gastrointestinal	33 (14.3)	35 (19.1)	0.193
**SOFA Score, median (IQR)**	1 (0, 2)	1 (0, 2)	**0.022**

*Data are n (%) unless specified otherwise. P < 0.05 was considered statistically significant. COVID-19, coronavirus disease 2019; IQR, interquartile range; COPD, chronic obstructive pulmonary disease; NS, no significance; DM, diabetes mellitus; SOFA, Sequential Organ Failure Assessment. Bold value means P < 0.05*.

### Laboratory Results

Some laboratory results at admission showed significant differences between male and female patients (*P* < 0.05). Male cases had substantially increased hemoglobin, alanine amino transferase (ALT), creatinine, creatine kinase, creatine kinase isoenzyme-MB (CK-MB), and procalcitonin, while significantly decreased lymphocyte counts, platelet counts, total cholesterol (TC) and high-density lipoprotein (HDL). Compared to men, women have lower scrum high sensitive c reaction protein (HS-CRP) levels (*P* < 0.001). The sex-specific laboratory results of the 413 patients with COVID-19 were shown in Table 2.

**Table 2:** Sex-specific laboratory results of COVID-19 patients.

**Variables**	**Normal Range**	**Male (*n* = 230)**	**Female (*n* = 183)**	***P*-value**
White blood cell (×10^9^/L)	3.5–9.5	7.90 (6.68, 10.2)	7.61 (6.54, 9.81)	0.172
Neutrophil (×10^9^/L)	1.8–6.3	6.13 (4.90, 8.37)	5.98 (4.67, 8.55)	0.434
Lymphocyte (×10^9^/L)	1.1–3.2	1.23 (1.0, 1.62)	1.36 (1.11, 1.72)	**<0.001**
Hemoglobin (g/L)	316–354	130 (123, 139)	117 (108, 124)	**<0.001**
Platelet (×10^9^/L)	125–350	251 (220, 313)	265 (233, 313)	**0.030**
D-dimer (μg/ml)	0–1.5	0.80 (0.43, 2.06)	0.92 (0.43, 2.25)	0.483
ALT (U/L)	9–50	36 (22, 53)	22 (15, 35)	**<0.001**
LDH (U/L)	120–250	344 (286, 446)	334 (278, 419)	0.165
IL-6 (pg/ml)	0–7	8.34 (6.08, 11.6)	8.47 (6.59, 10.6)	0.316
Prealbumin (mg/L)	200–430	125 (77, 169)	122 (83, 176)	0.842
Albumin (g/L)	40–55	30.2 (27.0, 33.8)	30.6 (27.3, 34.9)	0.184
Bilirubin (μmol/L)	0–26	12.0 (9.28, 17.2)	11.8 (9.15, 15.3)	0.316
Creatinine (μmol/L)	57–97	77.6 (68.6, 91.7)	56.9 (50.9, 67.0)	**<0.001**
Creatine kinase (U/L)	0–190	96.0 (62.3, 174)	58.0 (38.5, 95.0)	**<0.001**
CK-MB (U/L)	0–24	15.0 (11.0, 19.0)	13.0 (10.0, 16.0)	**<0.001**
HS-CRP (mg/L)	0–3	55.8 (19.3, 115)	25.3 (5.28, 60.9)	**<0.001**
Procalcitonin (ng/mL)	0–0.05	0.05 (0.05, 0.14)	0.05 (0.05, 0.07)	**<0.001**
Troponin (pg/ml)	0–28	4.95 (2.00, 15.2)	4.90 (2.0, 14.8)	0.756
TC (mmol/L)	3.3–5.2	3.52 (3.03, 4.13)	3.79 (3.26, 4.35)	**0.003**
HDL (mmol/L)	1.29–1.55	0.86 (0.70, 1.02)	1.02 (0.84, 1.20)	**<0.001**
LDL (mmol/L)	2.1–3.37	2.06 (1.62, 2.55)	2.05 (1.61, 2.55)	0.849
TG (mmol/L)	0.51–1.70	1.69 (1.49, 2.06)	1.77 (1.55, 2.17)	0.051
ESR (mm/H)	0–15	47.4 (32.8, 63.0)	51.5 (38.5, 67.4)	0.110

*Data are median (IQR). P < 0.05 was considered statistically significant. COVID-19, coronavirus disease 2019; ALT, alanine amino transferase; LDH, lactate dehydrogenase; IL-6, interleukin-6; CK-MB, creatine kinase isoenzyme-MB; HS-CRP, high sensitive c reaction protein; TC, total cholesterol; HDL, high-density lipoprotein; LDL, low-density lipoprotein; TG, triglyceride; ESR, erythrocyte sedimentation rate. Bold value means P < 0.05*.

### Clinical Outcomes

**Figure 1:**
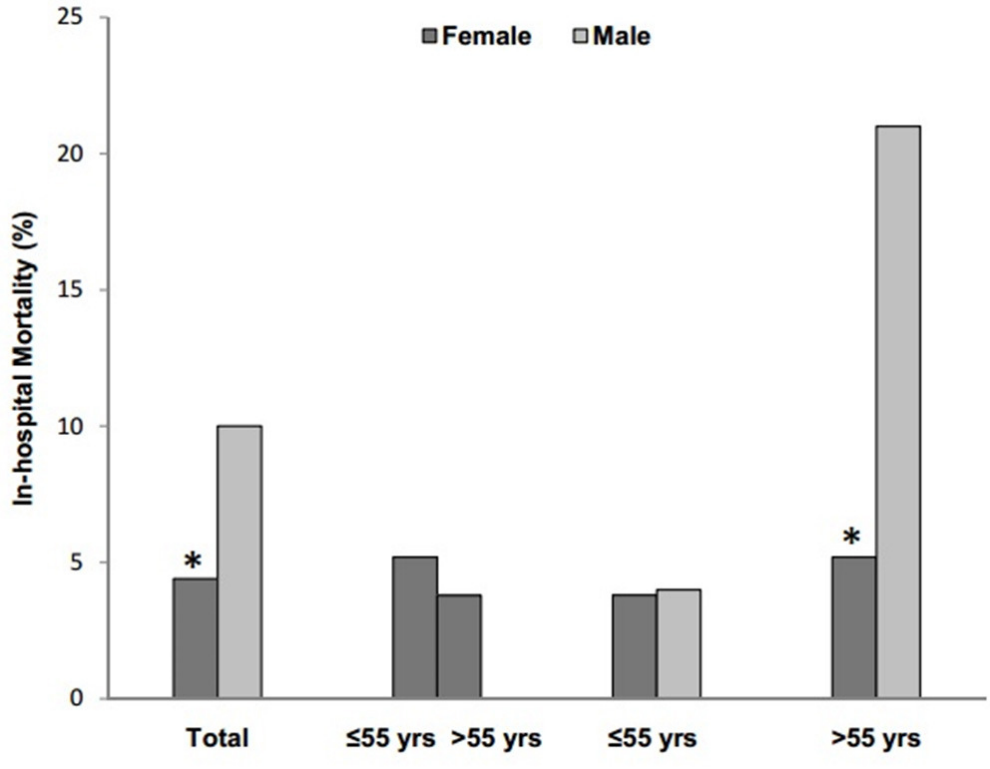
Sex-specific in-hospital mortality of COVID-19 patients and subgroup stratified by age of 55 years. * *P* < 0.05 vs. male by chi-squared tests or Fisher's exact tests.

**Figure 2:**
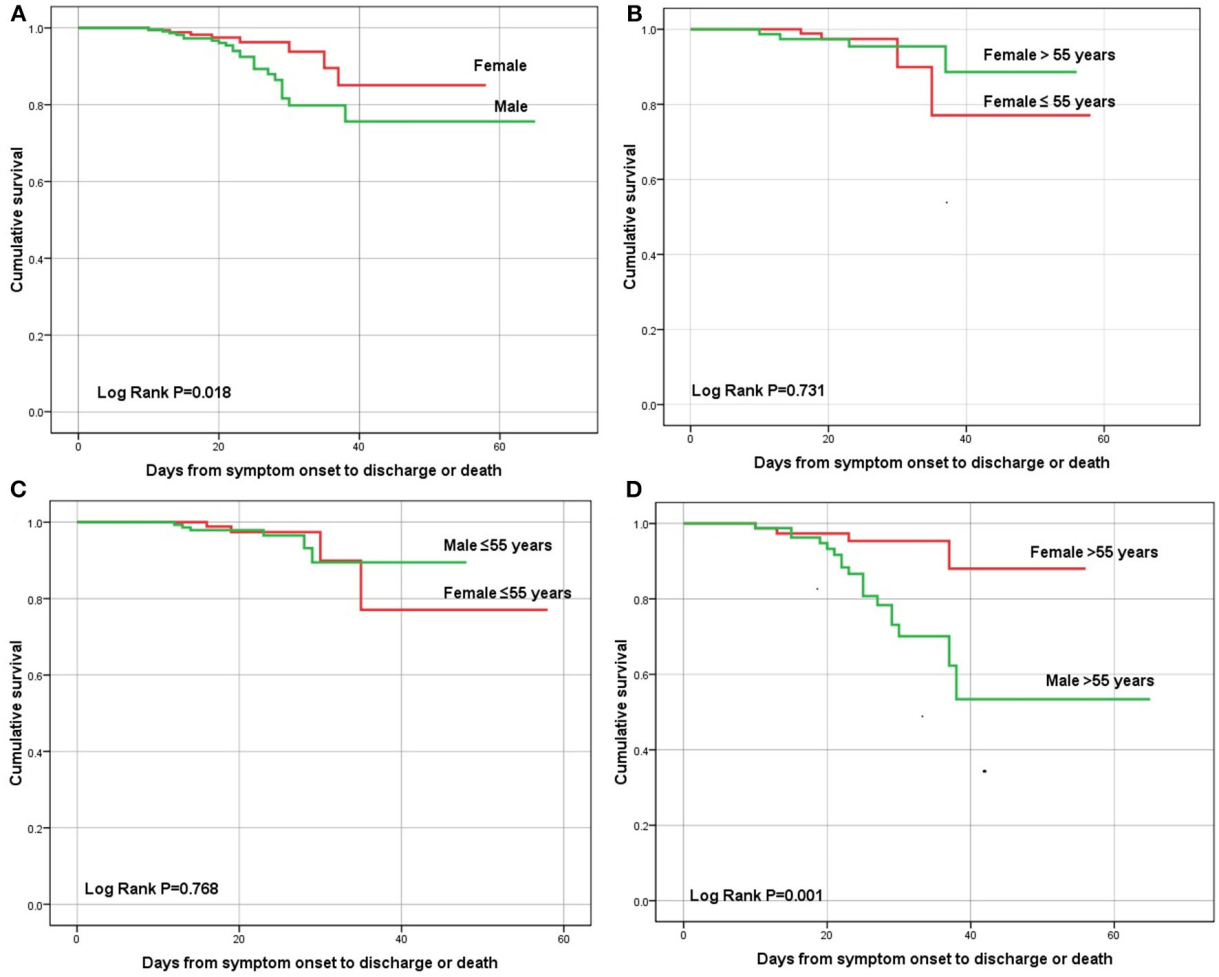
Kalpan-Meier survival curve of male and female patients with COVID-19 in **(A)** male vs. female, **(B)** age ≤ 55 years vs. age > 55 years in female patients, **(C)** male vs. female in age ≤ 55 years old group, and **(D)** male vs. female in age > 55 years old group by log-rank test for all.

The total of 31 patients (7.5%) died during hospitalization. Figure 1 showed the sex-specific mortality in different age patients. The in-hospital mortality rate was 10.0% for men and 4.4% for women (*P* = 0.031). The cumulative survival rate was significantly different between males and females (*P* = 0.018, log-rank test; Figure 2A). In the overall population, 91 cases (22.0%) were diagnosed as severe condition. Although there was no significant difference in the severity of COVID-19 between males and females, severe cases were more likely to be seen in men than women (24.3 vs. 19.1%, *P* = 0.203). There were no sex differences in development of ARDS, sepsis shock, acute kidney injury or acute liver injury (Table 3). However, compared with males, females were less likely to develop acute cardiac injury (6.6 vs. 13.5%, *P* = 0.022, Table 3).

**Table 3:** Sex-specific clinical outcomes of COVID-19 patients.

**Clinical outcomes**	**Male (*n* = 230)**	**Female (*n* = 183)**	***P*-value**
Diease severity			
Non-severe	174 (75.7)	148 (80.9)	–
Severe	56 (24.3)	35 (19.1)	0.203
ARDS	62 (27.0)	40 (21.9)	0.233
Time from symptom onset to ARDS, Median(IQR),d	11 (8, 16)	12 (8, 15)	0.748
Sepsis shock	22 (9.6)	12 (6.6)	0.269
Time from symptom onset to shock, Median(IQR),d	18 (13, 24)	17 (13, 23)	0.810
AKI	18 (7.8)	10 (5.5)	0.343
Time from symptom onset to AKI, Median(IQR),d	20 (14, 25)	17 (13, 22)	0.440
Acute liver injury	33 (14.3)	18 (9.8)	0.166
Time from symptom onset to acute liver injury, Median(IQR),d	17 (11, 23)	15 (11, 19)	0.840
Acute cardiac injury	31 (13.5)	12 (6.6)	**0.022**
Time from symptom onset to acute cardiac injury, Median(IQR),d	17 (11, 23)	16 (11, 21)	0.906
In-hospital mortality	23 (10.0)	8 (4.4)	**0.031**

*Data are n (%) unless specified otherwise. P < 0.05 was considered statistically significant. COVID-19, coronavirus disease 2019; ARDS, acute respiratory distress syndrome; IQR, inter quartile range; AKI, acute kidney injury. Bold value means P < 0.05*.

### Multivariate Cox Regression of In-hospital Mortality

Age and sex had significant interaction for the in-hospital mortality (*P* < 0.001) (Table 4). Multivariate Cox regression analysis suggested increased in-hospital mortality was associated with age (HR 1.041, 95% CI 1.009–1.073, *P* = 0.012), male sex (HR 2.033, 95% CI 1.007–2.098, *P* = 0.010), the interaction between age and sex (HR 1.118, 95% CI 1.003–1.232, *P* = 0.018), and comorbidities (HR 9.845, 95% CI 2.280–42.520, *P* = 0.002) (Table 4).

**Table 4:** Cox regression models evaluating risk factors associated with in-hospital mortality in COVID-19 patients.

**Variables**	**Univariate Cox regression**		**Multivariate Cox regression** **Model 1**		**Multivariate Cox regression** **Model 2**	
	**HR (95% CI)**	***P*-value**	**HR (95% CI)**	***P*-value**	**HR (95% CI)**	***P*-value**
Age	1.054 (1.023–1.087)	**0.001**	1.056 (1.026–1.085)	**0.001**	1.041 **(**1.009–1.073)	**0.012**
Age >55 yrs (vs. Age ≤ 55 yrs)	3.755 (1.719–8.205)	**0.001**				
Sex						
Male (vs. female)	2.431 (1.061–5.570)	**0.031**	2.027 (1.010–2.043)	**0.028**	2.033 (1.007–2.098)	**0.010**
Age × Sex	1.610 (1.276–2.030)	**<0.001**	1.206 (1.002–1.358)	**0.026**	1.118 (1.003–1.232)	**0.018**
Comorbidities	30.727 (9.125–103.461)	**<0.001**			9.845 (2.280–42.520)	**0.002**
Median time from symptom onset to admission, d	0.672 (0.543–1.384)	0.396				
Lymphocyte	0.164 (0.052–0.518)	**0.002**				
HS-CRP	1.007 (0.992–1.023)	0.353				
Procalcitonin	1.151 (1.007–1.314)	**0.039**				
D-dimer	1.016 (0.991–1.040)	0.210				
Troponin	1.002(0.996–1.009)	0.487				
TC	0.889 (0.524–1.508)	0.663				
HDL	1.356 (0.365–5.041)	0.649				
SOFA	1.881 (1.546–2.289)	**<0.001**				

*P < 0.05 was considered statistically significant. COVID-19, coronavirus disease 2019; HS-CRP, high sensitive c reaction protein; TC, total cholesterol; HDL, high-density lipoprotein; SOFA, Sequential Organ Failure Assessment. Comorbidities were defined as having at least one of the followings before diagnosis of COVID-19: chronic obstructive; pulmonary disease, diabetes mellitus, hypertension, coronary heart disease, chronic kidney disease, chronic liver disease, stroke, tumor, autoimmune disease, and HIV infection. Bold value means P < 0.05*.

### Subgroup Analysis

As mentioned earlier, female patients were divided into premenopausal and postmenopausal groups at the age of 55, and subgroup analysis was performed at the cut-off point of 55 years old. No differences on in-hospital mortality were noted in premenopausal women compared with postmenopausal women (3.8 vs. 5.2%, *P* = 0.144, Figure 1). In addition, subgroup analysis revealed no differences in-hospital mortality were noted in premenopausal women compared with men (3.8 vs. 4.0%, *P* = 0.918), but differed significantly in postmenopausal women with men older than 55 (5.2 vs. 21.0%, *P* = 0.007). Additionally, no difference in cumulative survival rate was noted in premenopausal women compared with postmenopausal women (*P* = 0.731, log-rank test; Figure 2B), as well as premenopausal women compared with men younger than 55(*P* = 0.768, log-rank test; Figure 2C), but differed significantly in postmenopausal women with men older than 55(*P* = 0.001, log-rank test; Figure 2D).

In the subgroup analysis, acute liver injury and acute cardiac injury were less observed complications in women than men older than 55 (10.4 vs. 24.7%, *P* = 0.019; 9.1 vs. 25.9%, *P* = 0.006, respectively). However, the median duration from onset of symptoms to complications had no difference between sexes. Compared with male patients, female patients had higher lymphocyte (*P* < 0.001) and high-density lipoprotein (*P* < 0.001), lower high sensitive c reaction protein level (*P* < 0.001), and lower incidence rate of acute cardiac injury (6.6 vs. 13.5%, *P* = 0.022). The sex differences of clinical characteristics and outcomes were shown in Tables 5–7.

**Table 5:** Sex-specific demographic and clinical characteristics for subgroup stratified by 55 years in COVID-19 patients.

**Variables**	**Age** **≤** **55 Y (*****n*** **=** **255)**			**Age** **>** **55 Y (*****n*** **=** **158)**		
	**Male (*n* = 149)**	**Female (*n* = 106)**	***P*-value**	**Male (*n* = 81)**	**Female (*n* = 77)**	***P*-value**
Age, Median(IQR), y	47 (37, 51)	46 (38, 50)	0.494	66 (61, 71)	66 (62, 73)	0.280
current or ever smoking	5 (3.4)	0 (0)	0.078	13 (16.0)	0 (0)	**<0.001**
Drinking	6 (4.0)	0 (0)	**0.043**	9 (11.1)	0 (0)	**0.003**
**Comorbidities**						
COPD	1 (0.7)	0 (0)	NS	5 (6.2)	1 (1.3)	0.236
DM	7 (4.7)	4 (3.8)	0.720	23 (28.4)	20 (26.0)	0.733
Hypertension	13 (8.7)	6 (5.7)	0.358	57 (70.4)	41 (53.2)	**0.027**
Heart disease	3 (2.0)	2 (1.9)	0.943	12 (14.8)	9 (11.7)	0.563
Kidney disease	1 (0.7)	1 (0.9)	NS	5 (6.2)	3 (3.9)	0.772
Liver disease	2 (1.3)	2 (1.9)	NS	5 (6.2)	3 (3.9)	0.772
Shock	1 (0.7)	0 (0)	NS	7 (8.6)	6 (7.8)	0.846
Tumor	1 (0.7)	1 (0.9)	NS	5 (6.2)	6 (7.8)	0.689
Immune disease	2(1.3)	2(1.9)	NS	2(2.5)	2(2.6)	NS
**Symptoms**						
Fever	141 (94.6)	86 (81.1)	**0.001**	67 (82.7)	64 (83.1)	0.947
Cough	114 (76.5)	82 (77.4)	0.874	64 (79.0)	60 (77.9)	0.868
Expectoration	56 (37.6)	32 (30.2)	0.221	25 (30.9)	25 (32.5)	0.829
Chest distress	62 (41.6)	43 (40.6)	0.867	48 (59.3)	49 (63.6)	0.572
Chest pain	3 (2.0)	4 (3.8)	0.646	2 (2.5)	4 (5.2)	0.632
Hemoptysis	2 (1.3)	1 (0.9)	NS	2 (2.5)	1 (1.3)	NS
Headache	9 (6.0)	7 (6.6)	0.855	3 (3.7)	4 (5.2)	0.945
Myalgia	15 (10.1)	13 (12.3)	0.580	9 (11.1)	11 (14.3)	0.549
Fatigue	53 (35.6)	32 (30.2)	0.369	30 (37.0)	39 (50.6)	0.085
Gastrointestinal	19 (12.8)	17 (16.0)	0.458	14 (17.3)	18 (23.4)	**<0.001**
SOFA Score, median (IQR)	1 (0, 2)	1 (0, 2)	0.189	2 (1, 3)	1 (0, 2)	**0.009**

*Data are n (%) unless specified otherwise. P < 0.05 was considered statistically significant. COVID-19, coronavirus disease 2019; IQR, interquartile range; COPD, chronic obstructive pulmonary disease; NS, no significance; DM, diabetes mellitus; SOFA, Sequential Organ Failure Assessment. Bold value means P < 0.05*.

**Table 6:** Sex-specific laboratory results for subgroup stratified by 55 years in COVID-19 patients.

**Variables**	**Normal range**	**Age** **≤** **55 Y (*****n*** **=** **255)**			**Age** **>** **55 Y (*****n*** **=** **158)**		
		**Male (*n* = 149)**	**Female (*n* = 106)**	***P*-value**	**Male (*n* = 81)**	**Female (*n* = 77)**	***P*-value**
White blood cell (×10^9^/L)	3.5–9.5	7.77 (6.77, 10.2)	7.45 (6.55, 9.10)	0.341	7.98 (6.76, 11.1)	7.76 (6.71, 9.51)	0.346
Neutrophil (×10^9^/L)	1.8–6.3	5.93 (4.95, 7.90)	5.89 (4.65, 8.03)	0.713	6.27 (4.87, 8.45)	6.00 (4.68, 8.60)	0.464
Lymphocyte (×10^9^/L)	1.1–3.2	1.35 (1.07, 1.77)	1.45 (1.13, 1.74)	0.532	1.13 (0.84, 1.40)	1.31 (1.10, 1.67)	**<0.001**
Hemoglobin (g/L)	316–354	135 (128, 141)	117 (108, 124)	**<0.001**	126 (115, 135)	116 (108, 124)	**<0.001**
Platelet (×10^9^/L)	125–350	274 (225, 349)	262 (234, 311)	0.536	240 (216, 286)	268 (232, 314)	**<0.001**
D-dimer (μg/ml)	0–1.5	0.56 (0.31, 1.15)	0.62 (0.33, 1.17)	0.720	1.11 (0.68, 4.15)	1.25 (0.58, 3.85)	0.803
ALT (U/L)	9–50	37.0 (25.0, 55.0)	22.0 (14.3, 37.5)	**<0.001**	33.0 (19.5, 51.5)	22.0 (15.0, 35.0)	**<0.001**
LDH (U/L)	120–250	331 (284, 397)	327 (281, 404)	0.941	356 (290, 479)	338 (278, 429)	**0.041**
IL-6 (pg/ml)	0–7	7.92 (5.81, 10.5)	7.77 (6.20, 10.7)	0.466	8.93 (6.31, 12.9)	8.91 (6.73, 13.2)	0.616
Prealbumin (mg/L)	200–430	151 (117, 215)	137 (92.5, 194)	0.079	89.0 (56.8, 129)	109 (79.0, 155)	**0.003**
Albumin (g/L)	40–55	30.0 (26.8, 33.7)	30.9 (27.0, 35.4)	0.107	30.3 (27.0, 33.8)	30.4 (27.4, 33.9)	0.795
Bilirubin (μmol/L)	0–26	11.8 (8.83, 18.4)	11.3 (8.90, 14.9)	0.553	12.1 (9.63, 15.4)	12.2 (9.38, 16.1)	0.735
Creatinine (μmol/L)	57–97	75.9 (68.8, 84.9)	54.2 (50.2, 61.1)	**<0.001**	81.2 (68.3, 99.2)	60.0 (52.2, 69.2)	**<0.001**
Creatine kinase (U/L)	0–190	92.5 (58.5, 168)	51.0 (38.3, 84.0)	**<0.001**	103 (67.0, 174)	60.0 (39.0, 117)	**<0.001**
CK-MB (U/L)	0–24	15.0 (11.0, 18.0)	12.0 (9.50, 16.0)	**0.005**	15.0 (12.0, 19.0)	13.0 (10.0, 16.0)	**0.002**
HS-CRP (mg/L)	0–3	26.7 (11.88, 82.4)	12.9 (3.90, 39.3)	**<0.001**	93.6 (44.3, 131)	26.0 (10.1, 63.9)	**<0.001**
Procalcitonin (ng/mL)	0–0.05	0.05 (0.05, 0.09)	0.05 (0.05, 0.05)	**<0.001**	0.07 (0.05, 0.23)	0.05 (0.05, 0.10)	**0.001**
Troponin (pg/ml)	0–28	4.70 (1.78, 14.3)	4.10 (1.80, 13.1)	0.968	6.95 (2.25, 17.5)	5.80 (2.25, 17.4)	0.979
TC (mmol/L)	3.3–5.2	3.62 (3.15, 4.17)	3.64 (3.11, 4.21)	0.883	3.42 (2.93, 4.00)	3.88 (3.38, 4.46)	**<0.001**
HDL (mmol/L)	1.29–1.55	0.85 (0.71, 1.00)	1.00 (0.81, 1.19)	**<0.001**	0.87 (0.70, 1.04)	1.05 (0.85, 1.21)	**<0.001**
LDL (mmol/L)	2.1–3.37	2.32 (1.82, 2.66)	2.01 (1.58, 2.46)	**0.028**	1.85 (1.52, 2.35)	2.13 (1.67, 2.64)	**0.016**
TG (mmol/L)	0.51–1.70	1.73 (1.54, 2.15)	1.78 (1.59, 2.19)	0.444	1.65 (1.45, 1.99)	1.77 (1.52, 2.15)	**0.024**
ESR (mm/H)	0–15	46.7 (35.3, 61.5)	49.5 (29.2, 61.9)	0.725	53.5 (28.1, 63.0)	58.6 (43.5, 72.2)	0.065

*Data are median (IQR). P < 0.05 was considered statistically significant. COVID-19, coronavirus disease 2019; ALT, alanine amino transferase; LDH, lactate dehydrogenase; IL-6, interleukin-6; CK-MB, creatine kinase isoenzyme-MB; HS-CRP, high sensitive c reaction protein; TC, total cholesterol; HDL, high-density lipoprotein; LDL, low-density lipoprotein; TG, triglyceride; ESR, erythrocyte sedimentation rate. Bold value means P < 0.05*.

**Table 7:** Sex-specific clinical outcomes for subgroupstratified by 55 years in COVID-19 patients.

**Clinical outcomes**	**Age** **≤** **55 Y (*****n*** **=** **255)**			**Age** **>** **55 Y (*****n*** **=** **158)**		
	**Male (*n* = 149)**	**Female (*n* = 106)**	***P*-value**	**Male (*n* = 81)**	**Female (*n* = 77)**	***P*-value**
Disease severity						
Non-severe	128 (85.9)	93 (87.7)	–	46 (56.8)	55 (71.4)	–
Severe	21 (14.1)	13 (12.3)	0.672	35 (43.2)	22 (28.6)	0.055
ARDS	25 (16.8)	14 (13.2)	0.435	37 (45.7)	26 (33.8)	0.126
Time from symptom onset to ARDS, Median (IQR), d	9 (7, 12)	11 (7, 16)	0.181	13 (8, 17)	12 (8, 16)	0.484
Sepsis shock	1 0 (6.7)	7 (6.6)	0.973	12 (14.8)	5 (6.5)	0.092
Time from symptom onset to shock, Median (IQR), d	17 (11, 20)	23 (14, 33)	0.244	18 (14, 25)	18 (13, 23)	0.828
AKI	5 (3.4)	4 (3.8)	0.859	13 (16.0)	6 (7.8)	0.111
Time from symptom onset to AKI, Median (IQR), d	20 (13, 22)	22 (21, 23)	0.438	16 (13, 22)	18 (14, 26)	0.374
Acute liver injury	13 (8.7)	10 (9.4)	0.846	20 (24.7)	8 (10.4)	**0.019**
Time from symptom onset to acute liver injury, Median (IQR), d	13 (10, 16)	13 (8, 22)	0.657	16 (13, 22)	17 (12, 23)	0.972
Acute cardiac injury	10 (6.7)	5 (4.7)	0.505	21 (25.9)	7 (9.1)	**0.006**
Time from symptom onset to acute cardiac injury, Median (IQR), d	15 (10, 18)	18 (17, 21)	0.145	16 (11, 23)	14 (9, 22)	0.202
In-hospital mortality	6 (4.0)	4 (3.8)	0.918	17 (21.0)	4 (5.2)	**0.007**

*Data are n (%) unless specified otherwise. P < 0.05 was considered statistically significant. COVID-19, coronavirus disease 2019; ARDS, acute respiratory distress syndrome; IQR, inter quartile range; AKI, acute kidney injury. Bold value means P < 0.05*.

## Discussion

To our best knowledge, this is the first multicentre retrospective cohort study to analyze the sex and estrogen effect on the clinical characteristics and outcomes in adult patients with SARS-COV-2 infection, and the role of estrogen in COVID-19 development. Estrogen modulates immune function in females and may contribute to resistance against infection, while estrogen level is significant difference before and after menopause. To explore the beneficial effect of estrogen in female COVID-19 patients, we made the subgroup comparison according to the age of menopause in women. We documented that fever was more common in men cases, while digestive symptoms were less common. Men with COVID-19 were more prone to develop into the severe condition and die. The same trend was also found in Europe (31). Although no difference in-hospital mortality was noted in women under 55 compared with the same age men, there was significantly difference in women over 55 with men of the same age group, which may be associated with a lower inflammatory response in premenopausal women. However, differences in mortality between premenopausal and postmenopausal women was not significant, suggesting that female mortality in COVID-19 was lower than male might not be directly related to the effect of estrogen. Experimental data showed that male sex was an independent risk factor associated with refractory disease and death (12, 32, 33). We also found that gender-related lifestyle, more chronic diseases, lower lymphocytes on admission, more complications and dyslipidemia may result in higher mortality rate in older men than in older women. These findings contribute to the discussion of whether older male patients with SARS-COV-2 infection should be paid more attention.

It has been known that males and females differ in their susceptibility and response to viral infections, resulting in sex differences in incidence and disease severity (18, 34). The reduced susceptibility of females to viral infections could be attributed to the protection from X chromosome and sex hormones, which play an essential role in innate and adaptive immunity (35). SARS-CoV-2 uses ACE2 on pulmonary endothelium as an entry receptor, while the gene for the ACE2 is on the X chromosome (36), which may be the reason for the higher prevalence of COVID-19 in men than in women. In addition, in premenopausal women, estradiol produced by the ovary, is the estrogen in largest quantity (40–400 pg/mL) and most potent. Nevertheless, ovary almost stops producing estradiol after menopause, leading to estradiol level is significant reduced in postmenopausal women (<20 pg/mL), and no difference with men. In our study, the inflammatory marker HS-CRP was lowest in premenopausal women, while HS-CRP may result in cytokine storms and relate to disease severity and mortality (2, 6). Previous study showed hypopituitary women had decreased level of estrogen and increased level of CRP (37). Therefore, the lower morbidity and mortality of premenopausal women may be related to estrogen-mediated low inflammatory response. We also found that no difference in mortality among premenopausal and postmenopausal women. This suggested estrogen influenced the infection with SARS-COV-2 and pathogenesis of COVID-19, but might not be directly related to the lower mortality in women. However, our study had the small sample size, and the majority of younger patients are being non-severe COVID-19 condition and fewer died. Further investigation with larger –scale data is needed to assess the influence of estrogen on COVID-19 patients.

Although advancing age is associated with greater risk of death in both sexes, the male bias remains evident (15). Our study suggested that age, male sex and comorbidities were independently associated with in-hospital mortality, as well as sex and age had significant interaction for in-hospital mortality of COVID-19. The age-related sex differences in patients with COVID-19 were consistent with reported cases of seasonal and pandemic influenza A virus infections in Australia and Japan (38, 39). In the present study, we found the increasing mortality might be associated with higher rates of smoking, hypertension and complications in older men. Smoking rate is higher among men than women worldwide (40), consisting with the result in this study. Such behavior is associated with the risk of developing comorbidities. Simultaneously, smoking is related to higher expression of ACE2 and may be a risk facto rfor disease prevalence and severity (4, 40, 41), but no firm conclusions can be drawn. Hypertension was the most common chronic diseases, particularly in older male patients. Previous epidemiological data indicated an association between hypertension and severe disease or death from COVID-19 (7–9). Besides, the incidence of ARDS and other complications were higher in older men with COVID-19, especially acute liver injury and acute cardiac injury, which might also affect disease severity and prognosis.

In the older group, total lymphocyte count was significantly lower in men than in women. Lymphocytes play a decisive role in maintaining immune homeostasis and inflammatory response throughout the body. Previous studies had suggested that severe COVID-19 patients might have lymphocyte responses. A meta-analysis reported lymphopenia had a 3-fold higher risk of developing severe COVID-19, and lower lymphocyte counts was an effective biomarker in predicting the severity and prognosis in COVID-19 patients (42). Therefore, lymphopenia may be one potential mechanism of age-related sex differences.

In this study, dyslipidemia was found in older patients infected with SARS-COV-2. The TC, HDL, LDL, and TG levels in older male patients showed markedly decreases as compared to older female patients. This goes along well with previously data, that the LDL, HDL and TC levels in COVID-19 patients showed significant decreases at the time on admission (43). Lipids play a central role in viral infection, as they represent the structural foundations of cellular and viral membranes, while LDL levels inversely correlated to disease severities, which could be a predictor for disease progress and poor prognosis (43). The exact mechanisms of dyslipidemia in COVID-19 patients have remained unclear; however, there were several potential causes. Firstly, circulating lipid became oxidized in the inflammatory conditions, which resulted in the loss their protective functions and contributed to ongoing inflammation (44). Meanwhile, inflammation regulated lipoprotein metabolism indirectly via the cytokines, such as resulting in reduced expression and secretion of apoprotein of HDL, remodeling HDL-associated proteome, and promote HDL clearance from plasma (45, 46). Therefore, the measurement of the oxidized LDL and apoprotein of HDL can confirm these notions. Secondly, hepatocytes are the dominant cell type determining systemic LDL levels, and liver injury which is a common complication in older men, can affect the serum LDL levels. Thirdly, older male patients may have exacerbated inflammatory response, while systemic inflammation may accelerate clearance of LDL, which may partly explain the reduction in circulating LDL. Finally, estrogen levels in postmenopausal women affect the normal levels of blood lipids, which lead to increase TC, TG and LDL levels.

## Limitation

There are several limitations in this study. Firstly, it was a retrospective multicentre study, and there were probably a significant referral bias, recall bias and measurement bias. Besides, this is an observational study and its results are subject to unobserved confounding factors. Secondly, we did not measure estrogen level and collect the history of hormone replacement therapy. However, we refer to previous studies and perform the subgroup analysis based on the age of menopause, to investigate the effect of estrogen in morbidity and mortality in patients with COVID-19 for the first time. Thirdly, only the indexes on admission were selected for analysis, without dynamic monitoring. Further, large-scale clinical studies and basic research are needed to explore risk factors for individualized assessment. Finally, no power calculation was made for the study, so the study is an exploratory study and its results are subject to false positive error and should be interpreted with caution.

In conclusion, older age, male sex and comorbidities were independently associated with in-hospital mortality of COVID-19 patients. Moreover, sex and age are interactively associated with outcome of COVID-19. Although female mortality in COVID-19 is lower than male in this study, it might not be directly related to the effect of estrogen. Further study is warranted to identify the sex difference in COVID-19 and mechanisms involved.

## Data Availability Statement

The original contributions presented in the study are included in the article/supplementary material, further inquiries can be directed to the corresponding author/s.

## Ethics Statement

The studies involving human participants were reviewed and approved by Jinan Infectious diseases Hospital, Shandong Provincial Chest Hospital, and Huanggang Central Hospital. The patients/participants provided their written informed consent to participate in this study.

## Author Contributions

YC and MM were involved in study concept and design and revised the final manuscript. JS, GQ, WS, CW, ZZ, XW, and XB collected the epidemiological and clinical data. PW, QY, and JJ processed statistical data. JS and GQ drafted the manuscript. All authors agree to be accountable for all aspects of the work in ensuring that questions related to the accuracy or integrity of any part of the work are appropriately investigated and resolved.

## Conflict of Interest

The authors declare that the research was conducted in the absence of any commercial or financial relationships that could be construed as a potential conflict of interest.
